# Retinal detachments in a patient with minimal change nephrotic syndrome: Case report and review of the literature

**DOI:** 10.3389/fneph.2022.1070792

**Published:** 2023-01-18

**Authors:** Chunjian Ye, Miaohua Qiu, Yu Zhong, Jiejian Chen

**Affiliations:** Department of Nephrology, The 900th Hospital of Joint Logistic Support Force, People's Liberation Army of China (PLA), Fuzhou, Fujian, China

**Keywords:** minimal change disease, retinal detachment, nephrotic syndrome, edema, hypoproteinemia

## Abstract

**Background:**

To report an unusual case of minimal change nephrotic syndrome with sudden bilateral retinal detachment.

**Case presentation:**

A 54-year-old woman with minimal change nephrotic syndrome presented with sudden-onset visual blurring in both eyes. Optical coherence tomography scans revealed macular schisis and extramacular intraretinal separation. A kidney biopsy confirmed the diagnosis of minimal change disease. Glucocorticoid therapy was quickly started. During remission, her vision was restored, with complete resolution of the subretinal fluid observed on optical coherence tomography.

**Conclusions:**

In minimal change nephrotic syndrome, fluid accumulation in the retina layer may occur, and gravity-induced vitreous traction on the inferior retina may cause retinal detachment. Patients should be advised to avoid large swings of the head and neck, handstands, and other activities that may increase the risk of retinal detachment. The possibility of retinal detachment should be considered when blurred vision occurs.

## Introduction

Eye problems associated with kidney disease are common in cases of autoimmune diseases or hereditary nephropathy (1–3), but ocular abnormalities associated with nephrotic syndrome are rarely reported (4–7). Minimal change nephrotic syndrome (MCNS) is characterized by gross proteinuria and severe hypoalbuminemia associated with rapidly progressive edema and often serous exudates. We present a case of MCNS complicated by acute serous retinal detachment in both eyes.

## Case report

A 54-year-old female dancer was admitted to our center, presenting with edema in both lower limbs, profuse proteinuria, and hypoalbuminemia. On admission, the patient’s height was 166 cm and her weight was 62 kg, which increased by 6 kg in 10 days. Her blood pressure was 108/74 mmHg. Urinalysis revealed high total protein levels (5.9g/24 h) without hematuria, a serum creatinine level of 72.4 μmol/l, a serum albumin concentration of 20.7 g/l, and a total cholesterol concentration of 14.7 mmol/l, and autoantibodies such as ANA and ANCA were negative. Various viral tests were also negative; there were no decline in complement C3 or C4, and the glycated hemoglobin level was 5.7%.

**Figure 1:**
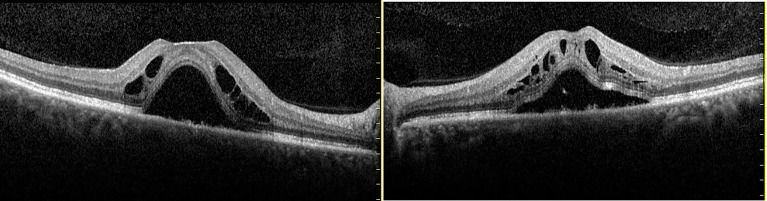
Optical coherence tomography scans of both eyes of a 54-year-old female patient who presented with decreased visual acuity in both eyes: (left) the right eye; (right) the left eye. Optical coherence tomography showed macular schisis and extramacular intraretinal separation. Alterations of the external limiting membrane, ellipsoid zone, and interdigitation zone were also found in both eyes.

On the 5th day of hospitalization, an ultrasound-guided renal biopsy was performed. After 2 days, based on professional habits, the patient conducted daily training. During the process, the patient suddenly experienced bilateral blurred vision. We applied for an ophthalmic consultation for her, and ocular evaluations were as follows: the visual acuity of the left eye was 0.4 and of the right eye was 0.4. Optical coherence tomography indicated bilateral serous retinal detachment, with retinal interlaminar cystoid changes in both eyes (**Figure 1**). Her daily training was suspended. Anticoagulant therapy with low-molecular-weight heparin was administered. Furosemide was administered, accompanied by salt and fluid intake restriction to control systemic edema.

**Figure 2:**
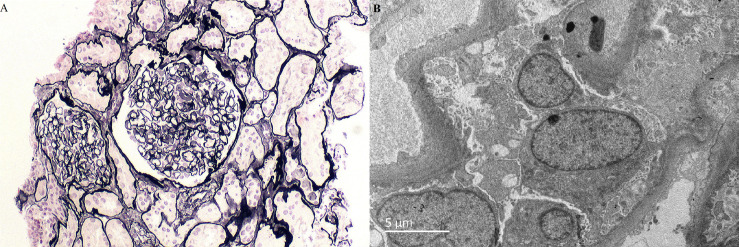
Light microscopy and electron microscopy images of histological changes in the renal biopsy. **(A)** Periodic acid–silver methenamine (PASM) (× 400). Mild glomerular lesions, no segmental sclerosis, tubular atrophy, and interstitial fibrosis. **(B)** Electron micrograph (× 6000) showed a normal glomerular basement membrane, no immune deposits, and widespread fusion of the epithelial cell foot processes.

**Figure 3:**
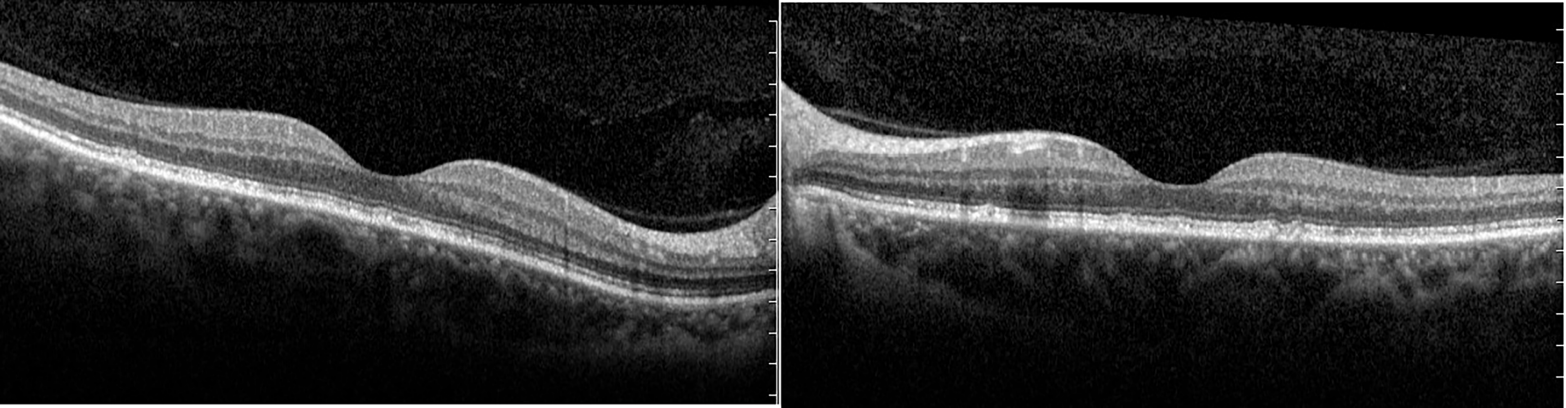
Optical coherence tomography scans of both eyes when partial remission was achieved, and visual acuity improved: (left) the right eye; (right) the left eye. Optical coherence tomography showed that serous retinal detachment had resolved completely and the macular area was equatorial in both eyes.

On the 10th hospital day, light microscopy of a renal biopsy specimen showed no significant glomerular abnormalities. Immunohistochemistry showed that there was no significant deposition of IgA, IgG, IgM, C3c, C3d, or C1q. Electron microscopy revealed extensive foot process effacement, and no electron-dense depositions, with no basement membrane abnormalities (**Figure 2**). We diagnosed her with minimal change disease. We administered 40 mg/day of methylprednisolone. From admission, her urine volume was about 900–1500 ml per day, her body weight increased from 62 to 64 kg, and there was no significant improvement in the edema or blurred vision. From the 14th day of hospitalization, her urine volume increased to over 2500 ml, even without diuretics. Edema resolved after a weight loss of 8 kg, and her visual acuity improved markedly to 0.8 in both eyes. In addition, optical coherence tomography showed that bilateral serous retinal detachment had resolved completely, the macular area was equatorial, and there was no obvious retinal detachment (**Figure 3**). The glucocorticoid regimen was changed to oral prednisone. The patient achieved complete remission 2 weeks after discharge, with no relapse in the 2 years following her first visit. The patient is still engaged in the dance profession and maintains daily training, and there has been no blurring of vision.

## Discussion

Edema in patients with nephrotic syndrome has been thought to be caused by two major factors which lead to retention: (1) primary sodium retention caused by kidney disease and (2) secondary sodium retention in which hypoalbuminemia reduces the plasma colloid osmotic pressure and the exudation of body fluid into the tissue space, leading to insufficient filling of the vascular system and activating the renin–angiotensin–aldosterone system (8). Retinal and choroidal thickening can also occur in the above pathophysiological process (9), resulting in macular edema and serous retinal detachment (4). The incidence of nephrotic syndrome complicated by retinal detachment is unknown; considering the relatively high incidence of nephrotic syndrome and the prevalence of tissue edema caused by nephropathy, and that there are few reports of retinal detachment, we suggest that nephropathy-related edema occurring in the retina and choroid is not sufficient to cause retinal detachment.

This patient is a professional dancer, and her daily training includes a wide range of head and neck swinging movements; the centrifugal force produced by such movements, or gravity-induced vitreous traction on the inferior retina may be the cause of retinal detachment. Valerie et al. reported a case of retinal detachment secondary to inversion table therapy (10) whose pathogenesis may be similar to that of this patient. MCNS usually responds well to glucocorticoid treatment. Peripheral edema will disappear rapidly after remission, accompanied by the improvement of retinal detachment and visual acuity. When the pathology of the kidney is not clear, use diuretics to resolve edema; improvement of retinal detachment and recovery of vision are also possible (4).

In conclusion, retinal edema that may be caused by nephrotic syndrome increases the risk of retinal detachment. Therefore, patients with nephrotic syndrome should be advised to avoid large swings of the head and neck, handstands, and other activities that may increase the risk of retinal detachment. The possibility of retinal detachment should be considered when blurred vision occurs.

## Data availability statement

The original contributions presented in the study are included in the article/supplementary material. Further inquiries can be directed to the corresponding author.

## Ethics statement

Ethics review and approval were not required for the study of human participants, in accordance with the local legislation and institutional requirements. Written informed consent from the patient was not required to participate in this study, in accordance with the national legislation and the institutional requirements.

## Author contributions

All authors contributed to the study conception and design. Material preparation, data collection, and analysis were performed by CY, MQ, YZ, and JC. The first draft of the manuscript was written by JC and all authors commented on previous versions of the manuscript. All authors read and approved the final manuscript.
